# Engineering the mind-body medicine: making a case for a trauma-informed primary care system

**DOI:** 10.25122/jml-2025-0129

**Published:** 2025-12

**Authors:** Satish Boregowda, Inga Eanes, Rodney Handy

**Affiliations:** 1 School of Mechanical Engineering, Purdue University, West Lafayette, IN, USA; 2 Hope for Healing, Council Bluffs, IA, USA; 3 Department of Environmental and Occupational Health and Safety Sciences, East Tennessee State University, Johnson City, TN, USA

**Keywords:** adverse childhood experience, trauma, stress, robust design, systems medicine

## Abstract

This narrative study proposes an engineering framework to model a value-based, trauma-informed primary care system. It is based on the premise that effective patient outcomes could be achieved by screening for adverse childhood experiences (ACEs). The protocol involves the administration of the ACE survey and an in-person trauma evaluation by mental health professionals embedded within the primary care settings. The ACE evaluation is then followed by the collection of psychophysiological stress response data. Depending on the level of symptomatic somatization, patients are then referred to appropriate treatment modalities. An engineering-based robust design methodology is utilized to demonstrate a model of a trauma-informed primary care system. To be deployed, the proposed value-based systems model of medicine warrants further investigation with clinical and empirical studies.

## INTRODUCTION

**Figure 1:**
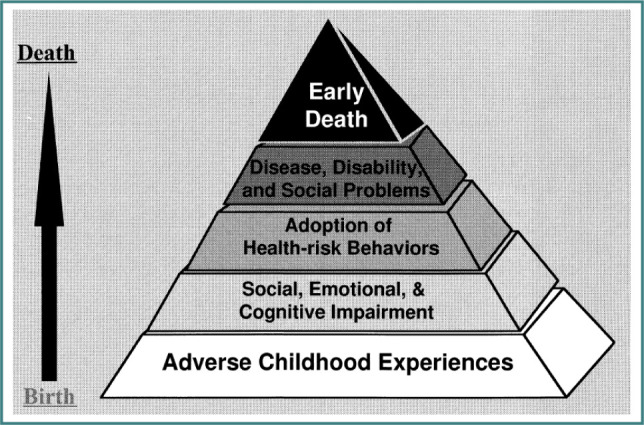
Potential influences throughout the lifespan of adverse childhood experiences (ACEs) [1]

The primary healthcare system is the entry point for all patients who exhibit a spectrum of physical symptoms resulting from acute to chronic illnesses. Some of these chronic illnesses include, but are not limited to, cancer, diabetes, heart conditions, autoimmune disorders, psychiatric conditions, and other related morbidities. The current system involves screening patients with results from pre-ordered laboratory tests and an office visit, followed by a referral to a specialist depending on the patient’s condition. While this system works, it falls short in terms of patient outcomes, cost, and utilization. In addition to physical examination of symptoms, it is critical to evaluate patients’ biopsychosocial history. It is hypothesized that the hidden biopsychosocial history plays a critical role in the development of symptoms leading to chronic illnesses. A large-scale (approximately 18,000 subjects) longitudinal study conducted by the Kaiser-Permanente/Center for Disease Control (KP/CDC) has established a strong correlation between adverse childhood experiences (ACEs) or household dysfunction and adult-onset chronic illnesses leading to early death, as shown in Figure 1. The ACE survey questions are provided in Appendix A, and the readers are encouraged to refer to the CDC/KP study [1] for further details.

Another study by Felitti [2] demonstrated that screening for ACEs, along with a detailed health appraisal process followed by appropriate therapeutic modalities, has contributed to reduced healthcare costs and utilization. It is suggested that 70% of diagnoses stem from the narrative patient history [3], which is captured in a 350-question questionnaire. While it is possible to implement this approach in an experimental setting, it would become cumbersome to deploy a 350-question health appraisal process in an operational primary care environment due to time and operational constraints. Furthermore, implementing this online might also lead to ethical, privacy, and technical data breach issues. Instead of a detailed health appraisal, it is easier to implement an in-person ACE evaluation. It has been shown that there is a relationship between ACEs and the victim’s self-perception and character [4]. With the help of this trauma narrative history, childhood information can be captured in an in-person interview by a mental health professional. The interview protocol is provided in Appendix B and can be further refined using the reference [5].

**Figure 2:**
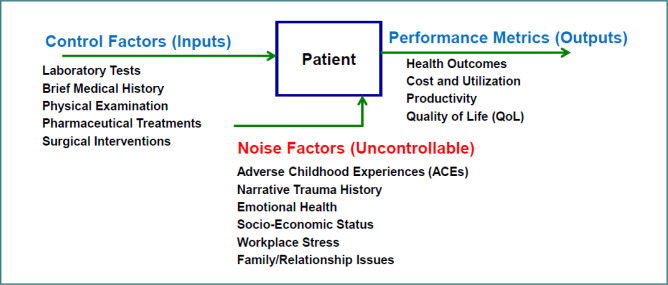
Parameter (P) diagram for trauma-informed primary care

This proposed systems model would support physicians and mental health professionals embedded within the primary care system working together as a team to address and treat the root causes of illness, rather than focusing on physical symptoms. A framework for implementing this holistic approach using a quality engineering-based robust design methodology is described in the next section.

### Robust design in the primary care system

A robust design methodology is a quality-engineering-based tool used in design and manufacturing to integrate quality into product design at an early stage of the production process. This is based on the premise that early intervention or prevention is better than cure, as advocated by the renowned quality management expert Edward Deming [6]. The production professionals on the factory floor who are well-informed about the supplier parts or raw materials can transform them into a valuable final product with less rework, scrap, and lower costs. By applying this value-based *Quality Management* principles to the healthcare system, the patients are analogous to machine parts or raw materials with detailed SOW (statement of work or work history) being supplied to the production personnel on the factory floor, who are like physicians, physician assistants, nurses, mental health professionals, and specialized physicians in the healthcare system [6,7]. In robust design, the control factors are the inputs to the system and are within the control of the physicians and other primary care health professionals. The ACEs, emotional health, workplace stress, and family and relationship issues are some of the noise factors that contribute to a larger portion of negative effects on the performance metrics or outputs (patient outcomes, cost, and utilization). The performance metrics (outputs) include health outcomes, cost, utilization, and productivity. These three factors are shown in the parameter diagram, commonly called the P-diagram, shown in Figure 2.

In this robust design, the word ‘parameter’ is equivalent to ‘factor’, and thus, we have the parameter (P) diagram. Several parameters (or factors) can influence the product’s quality characteristic or response [8,9]. With the implementation of robust design, physicians would work with supplier parts or materials (the patients in this case) with full information about their concealed defects, the hidden traumatic experiences.

## STRESS AND ADVERSE CHILDHOOD EXPERIENCE

**Figure 3:**
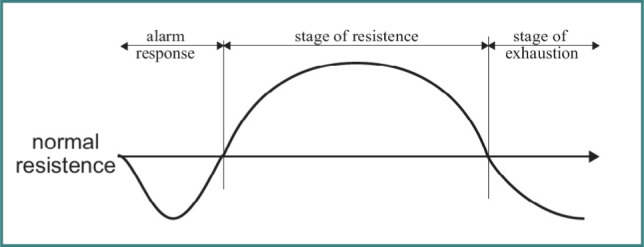
General adaptation syndrome (GAS) [8]

Stress has been defined as a nonspecific response of the body to any demand, and the ability of the body to mount a stress response without causing negative health effects requires adequate levels of adaptation energy [10]. Using the adaptation energy, the human physiological system adapts to stressful conditions, a process defined as the general adaptation syndrome (GAS), as illustrated in Figure 3.

There is a period of alarm response followed by a stage of resistance, resulting in a final stage of exhaustion. The nervous system responds to external stimuli by altering physiological responses. One’s ability to respond and overcome stressful events is controlled by the hypothalamic-pituitary-adrenal (HPA) axis, which plays a major role in triggering stress response and affecting numerous other bodily functions. This HPA axis is greatly affected by adverse childhood experiences (ACEs), which can deplete adaptive energy, thereby reducing the individual’s capacity to cope with daily stressors in both work and living environments. Furthermore, there has been a surge in studies examining the impact of childhood adversity on the HPA axis and individual stress reactivity. Several studies have shown a strong relationship between childhood trauma and the alteration of the HPA axis that, in turn, affects the stress response [11,12]. Several biomarkers of adverse childhood experiences (ACEs) are summarized in a study that included peripheral physiological indicators such as blood pressure and heart rate [13].

**Figure 4:**
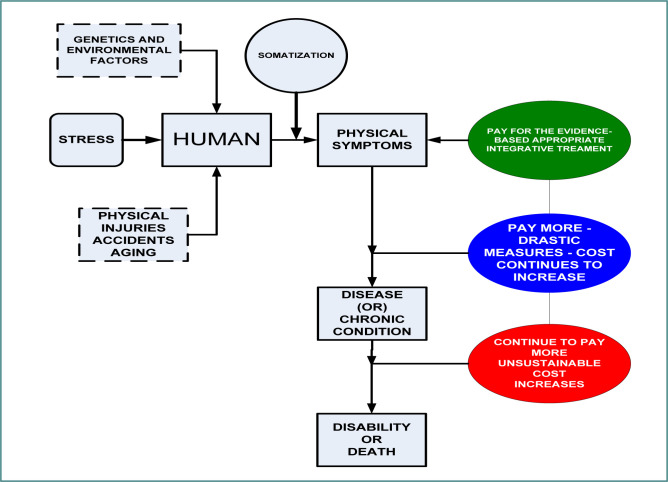
Stress and somatization in primary care patients

Furthermore, repeated activation of the HPA system increases cortisol production and, over time, negatively affects brain circuitry and brain volume development [14,15]. It has also been linked to dysregulation of blood pressure and blood sugar, as well as reduced cognitive functioning [16]. Just as the brain interprets our level of safety to generate an internal state of calm or an excited response that prompts the body to release more cortisol for energy, the process of discerning safety also involves an exchange of energy—information—between individuals and their environment, particularly with the people with whom children have relationships [17]. The quality of the energetic exchange in familial relationships is a critical element of the adverse childhood experience. Children are born into the world ready to connect. Children living in stressful environments absorb the emotional energy of their parents, who are authority figures [17]. This adaptation to survival in childhood leads to an altered vagus nerve, as explained in polyvagal theory [18]. The undiagnosed disorders and illnesses attributed to stress from ACEs lead to a degradation of human performance and ultimately workplace productivity, eventually resulting in huge economic losses [19]. Socio-economic and family system conditions dictate one’s physiological stress response to daily life stressors. The chronic psychological distress transforms into a physiological condition that later becomes a disease state, which is called *somatization* [20].It is critical to have a mental health system within the primary care to diagnose and treat patients at different levels of somatization [21]. Humans are complex systems characterized by biopsychosocial dynamics of both the present and the past. The lack of complete information regarding patient medical history and mental health status leads to ineffective treatments and subsequent suboptimal patient outcomes, increased costs and utilization, and loss of human productivity, contributing to major economic losses to society, as depicted in Figure 4.

## SYSTEMS MODEL OF MEDICINE

**Figure 5:**
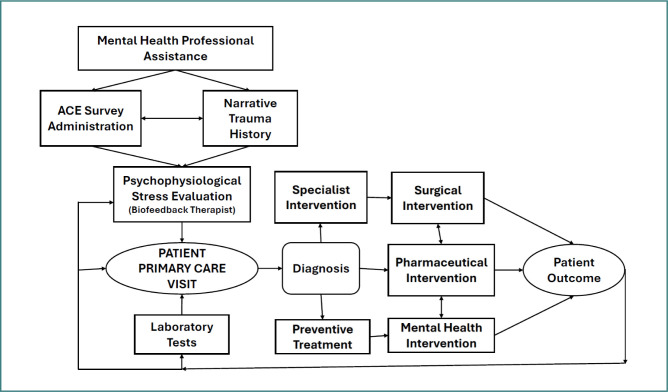
Systems model of primary care with embedded mental health professionals to diagnose and treat the effects of ACEs

The integration of mental health into primary care requires systems thinking [22]. This would provide a quantitative framework to better understand the impact of biopsychosocial factors on health and healing. Furthermore, systems thinking not only provides a quantitative framework for developing data-driven models but also aids in developing an operational plan to better integrate mental health processes within primary care, as shown in Figure 5. The proposed process involves the following three major steps: the collection of ACE survey data and a narrative trauma history by a mental health professional, and a psychophysiological stress evaluation by a mental health professional certified in biofeedback.

### Step I: Adverse Childhood Experience (ACE) Survey

The ACE questionnaire, shown in Appendix A, is administered by mental health professionals. Patients must trust and feel comfortable with their care providers during this process.

### Step II: Narrative trauma history

Mental health professionals collect a narrative trauma history of the patient in an in-person interview using the protocol in Appendix B. During this interview, patients would answer questions, interacting with social workers and/or clinical psychologists to do so in a safe environment. Patients should feel comfortable talking about their internal life struggles that are impacting their physiological conditions with mental health professionals more than with their physicians [23]. This is a very important step in diagnosing and treating a patient. In this regard, mental health professionals embedded within the primary care would play a critical role in transforming patient care.

### Step III: Psychophysiological stress evaluation

**Figure S1.**
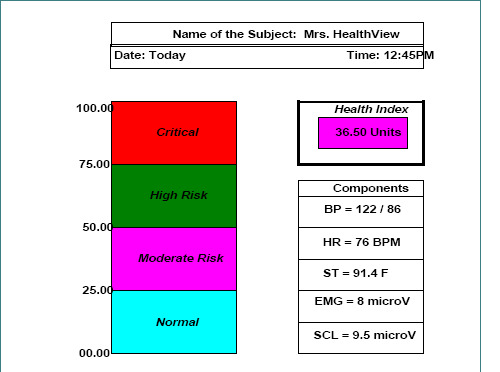
Psychophysiological stress evaluation system

Elevated stress levels lead to increased cortisol and other biochemical responses, resulting in cardiovascular metabolic dysregulation. This need needs to be quantitatively evaluated in terms of measurable peripheral physiological stress reactions in a medical setting. It involves collecting blood pressure, heart rate, finger skin temperature, electromyogram, and skin conductance level as shown in Appendix C (Figure S1) using the psychophysiological stress evaluation system. This step would be conducted by a mental health professional trained as a biofeedback therapist within the primary care setting. The protocol involves stress measurements at the end of three stages: relaxation (5 minutes), stressful Stroop task (10 minutes), and finally, recovery (5 minutes). Readers are encouraged to refer to the references [24,25] for details of the protocol.

## CONCLUSION

The current review article proposes a value-based systems model of medicine to integrate a three-step assessment of patients entering the primary care system. This approach involves an evaluation of adverse childhood experiences and psychophysiological stress evaluation by mental health professionals embedded within the primary care system. It has been well established in the studies cited in this paper that adverse childhood experience leads to adult-onset illnesses. Furthermore, it has been recognized that ongoing psychosocial stressors exacerbate the stress-related illnesses that lead to chronic health conditions. The treatment of these conditions requires a comprehensive evaluation of patients followed by a multitude of therapeutic approaches involving mental health professionals embedded within the primary care system. It is critical that a narrative trauma history of patients, in addition to the laboratory test results, is considered in tandem for the diagnosis and subsequent treatment of health conditions. A value-based systems model of medicine is proposed as a viable replacement to the existing fee-for-service biomedical model to reduce healthcare costs, improve quality of care, and achieve better patient outcomes. However, integrating mental health professionals and sophisticated computerized data-collection technologies into the primary care system could make value-based systems medicine a reality.

## Personal thanks

The authors wish to thank Dr. Vince Feletti for his encouragement and valuable suggestions to pursue this research project.

## APPENDIX C - Psychophysiological Stress Evaluation

Psychophysiological stress evaluation system was developed at NASA Laboratories for aerospace medical applications. The methodology involves the collection of multiple physiological signals - blood pressure, blood volume pulse, heart rate, finger skin temperature, electromyogram, and skin conductance level. Using this system, one could collect realtime stress response data in both work and living environments.

## References

[1] Felitti VJ, Anda RF, Nordenberg D, Williamson DF, Spitz AM, Edwards V (1998). Relationship of childhood abuse and household dysfunction to many of the leading causes of death in adults. The Adverse Childhood Experiences (ACE) Study. Am J Prev Med.

[2] Felitti VJ (2019). Health Appraisal and the Adverse Childhood Experiences Study: National Implications for Health Care, Cost, and Utilization. Perm J.

[3] Felitti VJ (2021). Private communication.

[4] Owusu J (2024). The impact of adverse childhood experience (ACE) on victim’s self-perception and character development in adulthood [dissertation].

[5] Creswell JW, Poth CN (2018). Qualitative inquiry and research design.

[6] Deming WE (1986). Out of the crisis.

[7] Taguchi G (1986). Introduction to quality engineering: designing quality into products and processes.

[8] Ulrich K, Eppinger S (2019). Product design and development.

[9] Phadke M (1989). Quality engineering using robust design.

[10] Selye H (1984). The stress of life.

[11] Heim C, Newport DJ, Mletzko T, Miller AH, Nemeroff CB (2008). The link between childhood trauma and depression: insights from HPA axis studies in humans. Psychoneuroendocrinology.

[12] Kern S, Laurent HK (2019). Childhood abuse predicts affective symptoms via HPA reactivity during mother–infant stress. Psychoneuroendocrinology.

[13] Deighton D, Neville A, Pusch D, Dobson K (2018). Biomarkers of adverse childhood experiences: a scoping review. Psychiatry Res.

[14] Siegel DJ (2013). Brainstorm: the power and purpose of the teenage brain.

[15] Harris NB (2018). The deepest well: healing the long-term effects of childhood adversity.

[16] Siegel DJ (2007). The mindful brain: reflection and attunement in the cultivation of well-being.

[17] Eanes I (2019). Relationship as an energetic exchange: a key theory for the nurtured heart approach [master’s thesis].

[18] Porges SW (2009). The polyvagal theory: new insights into adaptive reactions of the autonomic nervous system. Cleve Clin J Med.

[19] Heckman J, Knudsen E, Cameron J, Shonkoff J (2006). Economic, neurobiological, and behavioral perspectives on building America’s future workforce. Proc Natl Acad Sci U S A.

[20] Dickinson WP, Dickinson LM, deGruy FV, Candib LM, Main DS, Libby AM, Rost K (2003). The somatization in primary care study: a tale of three diagnoses. Gen Hosp Psychiatry.

[21] deGruy FV (1997). Mental healthcare in the primary care setting: a paradigm problem. Fam Syst Health.

[22] Meadows DH (2008). Thinking in systems.

[23] Goldstein E, Athale N, Sciolla AF, Catz SL (2017). Patient preferences for discussing childhood trauma in primary care. Permanente J.

[24] Boregowda SC, Downing BK, Handy R (2021). Thermodynamic evaluation of psychophysiological stress responses. J Hum Ergol.

[25] Boregowda SC, Handy R, Sleeth D, Riches N (2017). Thermodynamic degradation approach to quantify human stress response. J Thermodyn.

